# Exploring staff recruitment and retention in radiation therapy: global insights from an ESTRO RTT workshop

**DOI:** 10.1016/j.tipsro.2026.100412

**Published:** 2026-05-23

**Authors:** Sophie Perryck, Nigel Anderson, Philipp Scherer, Elizabeth Forde

**Affiliations:** a Department of Radiation Oncology, University Hospital Zürich, Zürich, Switzerland; b Icon Cancer Centres, South Brisbane, QLD, Australia; c Department of Medical Imaging and Radiation Sciences, Monash University, Clayton, Victoria, Australia; d University Clinic for Radiotherapy and RadioOncology of the PMU at the County Hospital Salzburg, Salzburg, Austria; e Applied Radiation Therapy Trinity, Discipline of Radiation Therapy, Trinity College Dublin, Ireland; f Trinity St. James’s Cancer Institute, Dublin, Ireland

**Keywords:** Workforce, Recruitment, Retention, Job satisfaction, Burnout, Resilience

## Abstract

Reduced recruitment and retention of radiation therapists (RTT) significantly impacts patient care and the profession. The European Society for Radiotherapy and Oncology RTT workshop explored professional motivation, early career experiences, progression and continuous education, and job satisfaction. Participants were initially motivated by working in a specialised, technology-driven field and making a meaningful patient impact, later shifting toward team-oriented goals. Addressing these evolving motivations is key to improving retention. Suggested strategies include fostering belonging during student placements, enhancing graduate onboarding, ensuring equal access to research and education funding, and enabling leadership autonomy to provide protected time from clinical duties.

## Highlights

• An ESTRO RTT Workshop identified workforce challenges facing our profession.
• Motivators to enter or leave the profession are similar globally.
• Dynamic strategies are needed to support staff as professional priorities evolve.

## Graphical abstract

**Figure:**
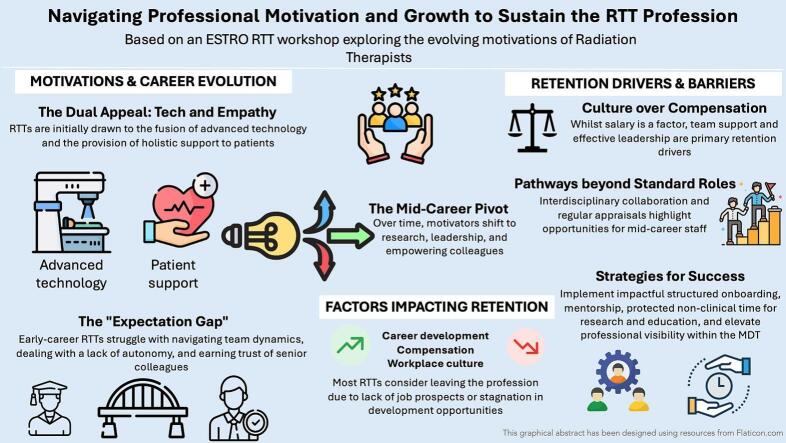

## Introduction

By 2050, cancer incidence is expected to increase by 77% from the 2022 estimate of 20 million, placing a significant burden on our workforce [1]. Zhu et al., [2] estimated that in 2050 the global radiotherapy workforce will require 141,077 radiation therapists (RTTs) in order to meet the workforce demand.

Staff recruitment and retention is a critical global issue in radiation therapy. Whilst a survey by the American Society of Radiologic Technologists demonstrate an increase in student enrolment into Radiation Therapy programmes [3], poor student attrition and staff retention have been acknowledged elsewhere [4], [5]. In the UK, RTT student attrition rate reportedly sits at approximately 25% [5], and only 22% of RTTs in New Zealand plan to remain in the profession until retirement age [6].

To better understand the human resource challenges facing our profession, a European Society for Radiotherapy and Oncology (ESTRO) workshop aimed to explore factors influencing recruitment and retention of RTTs, and to develop tangible solutions to support staff remaining in the profession. The paper reports on the process and outcomes of this workshop.

## Workshop process

The workshop was held across two online sessions in December 2024, and March 2025. Participants were invited to complete a pre-workshop survey to capture demographics and to probe initial impressions of four sub-themes

I) Exploring our motivators: Why become a Radiation Therapist?
II) Early career: expectations vs. reality
III) Education and Career Progression
IV) Professional challenges and workplace culture

Based on findings from the first session, eight challenges pertaining to recruitment and retention were identified (Table 1). The aim of the second session was to transition from professional challenges to possible solutions. In preparation, participants were invited to:

I) Complete a short survey reflecting on previous discussions
II) Proactively identify solutions which would be explored during the second session.
III) Prioritise the eight challenges previously identified
IV) Identify strategies to tackle their “top two” challenges

**Table 1:** Main challenges deduced from the first workshop session in the order that they were ranked during the second session.

Rank	Statement	Score
1	Job recognition (financial and personal)	5.70
2	Lack of support (time and money) for RTTs to engage in continuous education and research	5.50
3	Career transition − Keeping young colleagues motivated and help them find their field of interest / expertise.	5.20
4	Meeting Primary Motivations for becoming an RTT	4.30
5	Onboarding − Supporting students in becoming professionals/team members?	3.40
6	Specialisation − Can it actually limit career progression?	3.20
7	Educational programmes are no longer fit for purpose (do not reflect current practice)	3.10
8	Professional development (including additional education)	2.80

Completion of surveys and active participation in discussions was voluntary. Each workshop, including breakout rooms, was recorded, and transcribed with permission from all participants. Transcripts were summarised to capture key discussion points and identify areas of shared opinions. Microsoft Copilot was used as a supportive tool to assist with the initial summarisation of the transcripts, with all interpretations reviewed by the workshop faculty. Whilst the faculty contributed to the workshop surveys, and as such the quantitative findings, they did not actively contribute to the discussions. Potential bias was managed by limiting faculty to facilitation roles, standardising approaches through pre- and post-workshop meetings, and ensuring transparency via participant-led breakout summaries.

## Key insights

Nineteen professionals (including faculty) attended the workshops. The first pre-workshop survey was completed by 16 out of the 19 (84%) professionals. Participant characteristics can be found in Table 2.

Workshop Session 1: Motivators and Challenges

**Table 2:** Characteristics of the participants (n = 16) recorded with a pre-workshop questionnaire (supplementary material) as well as speciality and current role from the registration information (n = 19).

Participant characteristics	Category	n (%)
Speciality (participants only)	Radiation Therapist	18 (95%)
	Other	1(5%)

RTT role	Advanced practitioner	6 (32%)
	Educator	5 (26%)
	Manager	7 (37%)
	NAN	1 (5%)

Highest degree awarded	Doctorate	5 (31%)
	Masters	7 (44%)
	Undergraduate	3 (19%)
	Graduate Diploma	1 (6%)

Years experience (overall)	0–5 years	0 (0%)
	6–10 years	2 (13%)
	11–15 years	3 (19%)
	> 15 years	11 (69%)

Years experience (current role)	0–5 years	6 (38%)
	6–10 years	4 (25%)
	11–15 years	3 (19%)
	> 15 years	3 (19%)

### Exploring our motivators: Why become (and remain) a radiation Therapist?

When asked to reflect on why participants chose to become an RTT, common themes emerged. The integration of advanced technologies alongside patient care was universally appealing; a sentiment which many can identify with [7]. Participants felt radiotherapy presented an opportunity to utilise highly sophisticated systems, with frequent technological advancement, whilst also providing empathetic support to patients during a challenging time in their lives. An aspiration to simply help people and make a meaningful impact was a strong motivator. Pursuing a career as an RTT was thought by many to be extremely rewarding, working as part of a dynamic team, and the perceived opportunity for ongoing professional development was highly valued. Conversely, some participants did not possess the same level of knowledge or sense of purpose as some of their colleagues when entering the profession [5].

Nightingale et al., [8] demonstrated that perceptions and needs change across career trajectory. Similarly, participants collectively reported a motivational shift, i.e., what originally inspired them to enter the profession has now been replaced with newly identified motivators. Given the high percentage (69%) of participants who have worked as an RTT for > 15 years, it is not surprising that many have experienced a strong transition from direct patient care responsibilities to broader roles, such as leadership and management. With these changing roles, they now find fulfilment in empowering colleagues and enabling others to provide high-quality patient care rather than doing so directly themselves. Several participants reported that engaging in research, exploring innovation, and contributing to multidisciplinary projects have also become significant motivators over time.

### Early career: Expectations vs. Reality

Participants reflected on their transition from student to professional role. Using a 10-point Likert scale (1 = high alignment, 10 = poor), most reported moderate alignment between expectations and reality, scoring between 2 and 4. A key challenge was gaining confidence in independent decision making, seen as essential for professional growth. Another major hurdle was earning colleagues’ trust to work autonomously. Providing a strong sense of professional identity and exposure to a breadth of experiences is critical to supporting staff during this foundational period in their careers [9], [10]. Some also experienced a loss of autonomy when changing roles or countries, prompting them to pursue further education or role development.

Difficulties with interprofessional communication, team dynamics, and workplace politics led some to question their career choice. Limited opportunity for career advancement due to existing hierarchical structures and lack of professional recognition also prompted doubts over chosen profession. Emotional and ethical stress from treatment decisions and challenging interactions made the role overwhelming for some. Difficult transitions to practice were also linked to training differences and gaps between academic learning and clinical reality, often requiring additional upskilling or on the job training, both hard to achieve in a clinical setting.

The suitability of existing educational programmes has been previously explored within ESTRO. Where Campbell et al. [11] discuss curriculum gaps, this workshop has provided deeper insights to the impact of foundational education on job satisfaction; specifically highlighting the “reality shock” of moving from student to professional. Increased exposure to practical and clinical experiences is critical in bridging the gap between learning and practice [10].

### Education and career progression

In addition to undergraduate education, the impact of continuous professional development (CPD) on career progression was explored. It was unanimously acknowledged that pursuing CPD activities is costly and time consuming. Despite this, 56% of respondents indicated a need for additional education to keep up in a rapidly changing field (Supplementary Material). Networking and research collaboration opportunities were highly valued, reflecting a desire for interdisciplinary engagement. Most participants (69%) relied on international societies like ESTRO for education, while 50% used national universities and 44% national societies. Funding for continued education was available to 56%. While 69% had time off clinical duties for educational events, only 31% received study leave for research activities. Mid-career stagnation and limited progression paths were identified as challenges impacting staff retention, aligned with Nightingale et al [8]. Whilst some participants felt that advanced practice (AP) roles were not reflected in job progression, others felt there was simply a lack of clear progression paths at all. The lack of consensus on the definition of AP roles and the recommendation to develop guidelines under a European APRT framework detailing clear progression pathways had been highlighted in a previous ESTRO RTT workshop [12]. These mid-career professionals are at risk of being disengaged if they feel there is insufficient stimulation or meaningful collaborations within the interdisciplinary team, either locally or globally [13].

### Professional challenges and workplace culture

**Fig. 1:**
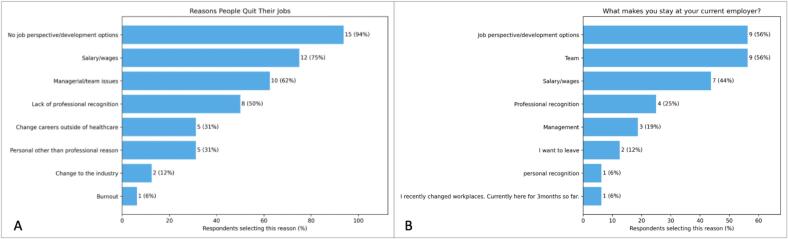
Participant perspectives on reasons people quit their job (A) and factors influencing respondents to stay with their current employer (B). Bars display the proportion of workshop survey respondents selecting each reason, calculated as the percentage of total respondents (n = 16). Due to multiple selections being allowed, percentages exceed 100% in total.

Ninety-four percent of participants felt people may feel compelled to leave the profession due to a lack of “job perspectives or development opportunities”, consistent with previous findings [14] (Fig. 1A). “Salary/wages” was the second highest contributing factor, with 75% of participants selecting this response. Taylor et al., [6] also explored this topic; however, salary was not a contributing factor, with RTTs in New Zealand rating their pay as “good” or “very good”. In our workshop, despite a more widespread understanding of the impact of professional burnout in recent years [15], [16], burnout was only indicated by one participant. During discussions it was noted, that leaving a job is not a decision people take lightly, and the reasons are most likely to be multifactorial.

**Fig. 2:**
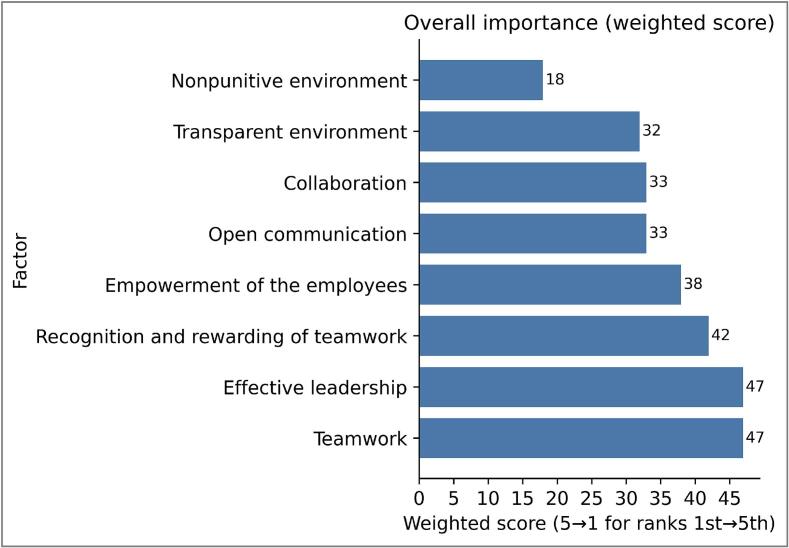
Horizontal bar chart showing the overall importance of factors contributing to a positive work environment, based on weighted scores derived from respondent rankings. Scores were calculated by assigning 5 points to factors ranked first, decreasing to 1 point for factors ranked fifth. Factors are ordered from highest to lowest weighted score.

Focusing on professional satisfaction, participants were also asked to consider what prompts them to stay with their current employer. Where “Salary/wages” scored highly as a driving factor for people seeking employment elsewhere, 44% of participants also cited it as a retention reason, indicating they are happy with their remuneration (Fig. 1B). Seven participants were managers and 69% had over 15 years’ experience, likely influencing salary perceptions. “Job perspective and development opportunities” were highlighted by 56%, reflecting the presence of APRTs and a highly educated group (75% with higher degrees. Again, the lack of more junior RTT workshop participants potentially influenced these findings. Team support was cited by 56% as key to retention, with teamwork and effective leadership ranking highest in contributing to a positive work culture (Fig. 2). A non-punitive environment ranked lowest, likely reflecting radiotherapy’s already strong safety culture, where open error and near miss reporting is standard.

Workshop Session 2: Strategies for Success

A pre-workshop survey combined with live polling identified three top priorities to support staff retention: providing clear career transition; addressing poor job recognition; and managing time constraints.

### Providing clarity around career transition

During the second session, the importance of valuing students during their placements to encourage future employment interest was emphasised. The difficult transition period from student to practitioner previously noted by Opoku et al., [10] and Matthews et al., [9] was also enthusiastically acknowledged in the workshop. One participant shared a successfully structured onboarding program when transitioning into the workforce, addressing common issues such as imposter syndrome or lack of confidence in decision making. The benefits of structured onboarding have already been demonstrated for nurses and physicians [17]. Mentorship and equal opportunities were highlighted as key motivators for early career RTTs, though only 31% reported access to a mentor or research supervisor. For mid-career staff, regular appraisals support discussion of professional goals. Expanding opportunities beyond standard roles and encouraging interdisciplinary collaboration can broaden perspectives and highlight pathways for career development and growth.

### The impact of professional identity and job recognition

Participants emphasised improving RTT visibility within multidisciplinary teams by promoting services and engaging in hospital committees. Suggested strategies included offering radiotherapy department tours to other disciplines and nominating RTTs for institutional awards. Recognising contributions through patient feedback was also seen as valuable, helping to boost professional pride and overall job satisfaction. Creating a positive workplace culture has been shown to benefit not only staff [15], but has also been found to be associated with improved patient outcomes [18].

Finally, providing high quality, radiotherapy specific information to both patients and the public via various media/channels was seen as one approach to enhance perceptions and understanding of our profession, and may have a positive impact on student registration and recruitment of new graduates, as demonstrated by Murphy et al [4].

### Protecting our time and providing equal opportunities

Providing funding for CPD and protected research time was seen as essential for retention, with gaps in both areas posing significant barriers. Funding challenges for postgraduate study and CPD vary globally, highlighting the role of national societies in offering affordable education and the potential of industry support. Regardless of funding, protected time for research and development is critical but difficult in stretched workforces, requiring managerial support. Dedicated non-clinical time, common in professions like nursing, was suggested. Clear career pathways that recognise qualifications and research are also important, as lack of financial or professional recognition can discourage further education.

## Conclusion

This ESTRO RTT workshop brought together international RTTs to explore motivation, early career experiences, CPD and factors affecting retention and job satisfaction. Key motivators and challenges were consistent across regions and align with existing literature. Improving recruitment and retention depends on fostering a positive workplace culture and a sense of belonging within the multidisciplinary team. Despite ongoing challenges RTTs remain driven by a shared commitment to delivering high-quality, patient centred care.

GenAI Statement

Microsoft Copilot was used as a supportive tool to assist with the initial summarisation of the recorded transcripts. Microsoft Copilot was also used in the creation of Fig. 1, Fig. 2. All AI-generated outputs were verified for accuracy by the authors.

## Funding

This research did not receive any specific grant from funding agencies in the public, commercial, or not-for-profit sectors.

## Declaration of competing interest

The authors declare that they have no known competing financial interests or personal relationships that could have appeared to influence the work reported in this paper.

## Appendix A Supplementary data

Horizontal bar chart incicating motivations for pursuing additional educational opportunities in radiation therapy. The following are the Supplementary data to this article:
