# Supplementary material for: Exploring staff recruitment and retention in radiation therapy: global insights from an ESTRO RTT workshop
Source: Tech Innov Patient Support Radiat Oncol. 2026 May 23;38:100412. doi: 10.1016/j.tipsro.2026.100412 (PMC13224374; doi:10.1016/j.tipsro.2026.100412)
Supplement: Supplementary Data 1 [file mmc1.docx]

**SUPPLEMENTARY MATERIAL**


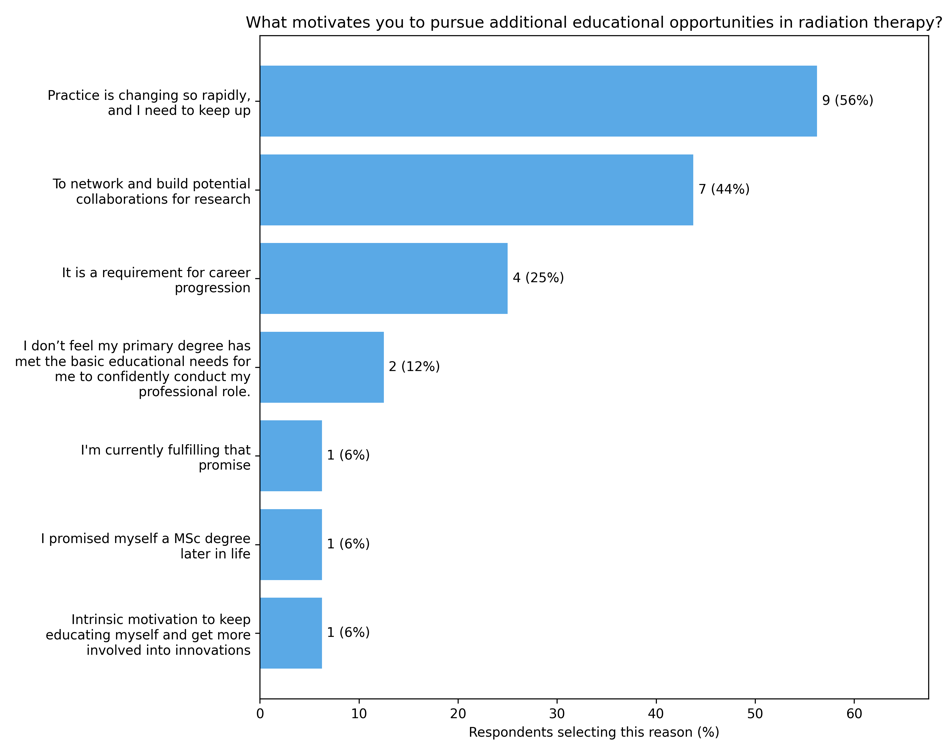


Figure S1: Motivations for pursuing additional educational opportunities in radiation therapy. Bars show the percentage of respondents selecting each option. Due to multiple selections being allowed, percentages exceed 100% in total.
